# IDO1 and IDO2 Non-Synonymous Gene Variants: Correlation with Crohn's Disease Risk and Clinical Phenotype

**DOI:** 10.1371/journal.pone.0115848

**Published:** 2014-12-26

**Authors:** Alexander Lee, Navya Kanuri, Yuanhao Zhang, Gregory S. Sayuk, Ellen Li, Matthew A. Ciorba

**Affiliations:** 1 Division of Gastroenterology, Washington University in St. Louis School of Medicine, St. Louis, Missouri, United States of America; 2 Department of Applied Mathematics and Statistics, Stony Brook University, Stony Brook, New York, United States of America; 3 Division of Gastroenterology, Veteran Affairs Medical Center, John Cochrane Division, St. Louis, Missouri, United States of America; 4 Division of Gastroenterology, Stony Brook University, Stony Brook, New York, United States of America; University of Chicago, United States of America

## Abstract

**Background:**

Crohn's disease (CD) is a chronic inflammatory disease of the gastrointestinal tract. Genetic polymorphisms can confer CD risk and influence disease phenotype. Indoleamine 2,3 dioxygenase-1 (IDO1) is one of the most over-expressed genes in CD and mediates potent anti-inflammatory effects via tryptophan metabolism along the kynurenine pathway. We aimed to determine whether non-synonymous polymorphisms in IDO1 or IDO2 (a gene paralog) are important either as CD risk alleles or as modifiers of CD phenotype.

**Methods:**

Utilizing a prospectively collected database, clinically phenotyped CD patients (n = 734) and non-IBD controls (n = 354) were genotyped for established IDO1 and IDO2 non-synonymous single nucleotide polymorphisms (SNPs) and novel genetic variants elucidated in the literature. Allelic frequencies between CD and non-IBD controls were compared. Genotype-phenotype analysis was conducted. IDO1 enzyme activity was assessed by calculating the serum kynurenine to tryptophan ratio (K/T).

**Results:**

IDO1 SNPs were rare (1.7% non-IBD vs 1.1% CD; p = NS) and not linked to Crohn's disease diagnosis in this population. IDO1 SNPs did however associate with a severe clinical course, presence of perianal disease, extraintestinal manifestations and a reduced serum K/T ratio during active disease suggesting lower IDO1 function. IDO2 minor allele variants were common and one of them, rs45003083, associated with reduced risk of Crohn's disease (p = 0.025). No IDO2 SNPs associated with a particular Crohn's disease clinical phenotype.

**Conclusions:**

This work highlights the functional importance of IDO enzymes in human Crohn's disease and establishes relative rates of IDO genetic variants in a US population.

## Introduction

Crohn's disease (CD) is a chronic inflammatory bowel disease (IBD) affecting of millions of people of all races worldwide. Current evidence suggests that CD occurs in genetically susceptible individuals who develop loss of tolerance and a resultant chronic immune response against commensal luminal microbiota, likely in response to an antecedent environment trigger. [1], [2] Genome wide association studies (GWAS) have identified over a hundred and sixty genetic loci and non-synonymous single nucleotide gene variants (SNPs) which associate with risk of developing CD. [3] Many of these genes relate to microbial defense mechanisms, epithelial barrier function and the innate and adaptive immune systems. [4] However, less than 15% of CD variance is explained by these genes [3] and many genes may impact disease phenotype or severity rather than influence disease risk.

Indoleamine 2,3 dioxygenase-1 (IDO1) is a widely expressed enzyme which is the initial and rate limiting step of tryptophan catabolism along the kynurenine pathway. IDO1 expression is inducible by inflammatory stimuli including cytokines and toll like receptor agonists. The resulting suppression of local tryptophan and increase in bioactive kynurenine pathway metabolites functions to reduce inflammation and promote immune tolerance via several mechanisms. [5] Among these include exertion of antimicrobial activity, suppression of activated T-cell responses and induction of regulatory T-cells. Thus, acting as a natural break to ongoing inflammation, it is not surprising that increased IDO1 expression has been identified in active IBD and CD. [6], [7] Mechanistic studies using experimental models have advanced our knowledge by revealing that inhibition of IDO1 leads to worsened colitis severity [8], while pharmacologic induction of IDO1 can limit colitis severity and promote epithelial restitution [9], [10].

Based on this experimental and observed human data, we hypothesized that individuals carrying a functionally relevant SNP of the IDO1 gene may exhibit unchecked inflammation and thus experience a more severe disease course if affected by Crohn's. Though not identified as such in GWAS studies to date, it is also possible that IDO1 SNPs may confer risk for development of CD in some populations. To address these hypotheses we examined a prospectively enrolled cohort of well-characterized CD patients and a non-IBD control cohort for known IDO1 SNPs. We also examined the same population for the variants of the more recently discovered gene analog of IDO1, IDO2. While its expression is more restricted than that of IDO1, its expression in the colon is reported. [9], [11] To estimate the relevance to enzyme function, we also compared the serum tryptophan to kynurenine ratio in patients with and without IDO1 gene variants.

## Methods

### Identification of IDO Variants

This protocol was approved by the Human Research Protection Office of Washington University School of Medicine and all clinical investigation was conducted according to the principles expressed in the Declaration of Helsinki. All participants provided their written informed consent to participate in this study. To identify nonsynonymous single nucleotide variants for IDO1 and IDO2 and their expected frequencies we used the online public databases HapMap [12] and dbSNP. [13] We also reviewed the literature to identify additional nonsynonymous SNP and non-single nucleotide variants. [14] For IDO1, six nonsynonymous variants were identified. Five of the six variants were SNPs: rs4463407, rs12545877, rs35059413, 35099072, and C-to-A in exon 7 (no rs number, designated 7CA); one of the six variants was a 9 base pair deletion in exon 7 (no rs number, designated 9BPD). For IDO2, five nonsynonymous variants were identified. All were SNPs: rs4503083, rs4736794, rs10109853, rs35212142, and rs35446289.

### Patients and Clinical Variables

All patients included in this study were prospectively enrolled by providing written informed consent as part of the Washington University in St Louis Division of Gastroenterology's Digestive Disease Research Cores Center (DDRCC) BioBank core. This repository included blood, saliva, and/or tissues for genotyping, obtained via recruitment in consecutive fashion during inpatient and outpatient visits as previously described. [15] The specimen repository is linked to a database containing demographic information and clinical history. Data was accessed from patients enrolled between May 2005 and January 2011. From this institutional cohort, we identified patients for inclusion in our study as all Crohn's disease subjects with DNA available for genotyping as well as with comprehensive clinical variables of interest available: birth date, age at diagnosis, gender, ethnicity, family history of IBD, history of IBD-related surgery, medication history and presence of extraintestinal manifestations of IBD. All CD patients were categorized by Montreal Classification [16] as part of the BioBank core intake assessment. The non-IBD controls included a validated cohort of individuals enrolled in the BioBank core either as healthy controls through a hospital wide recruitment process or via clinic or endoscopy appointments for non-IBD indications (commonly colorectal cancer screening). [17] A standard medical history and physical exam was used to exclude IBD or chronic inflammatory conditions and endoscopic substantiation was available in most cases. Patients were excluded only if there was inadequate material for genotyping and/or insufficient data for clinical variables of interest.

### Genotyping

Primer specific genotyping was performed by Sequenom MassARRAY (Sequenom Inc., San Diego, CA) as previously described by our group. [15] This approach involves PCR amplification of the region containing the SNP of interest, an optimized primer extension reaction to generate allele-specific DNA products, and chip-based mass spectrometry for separation and analysis of the DNA analytes. A single post-PCR primer extension reaction generates diagnostic products that, based on their unique mass values, allow discrimination between two alleles. Specifically, determination of mass relies on MALDI-TOF (matrix-assisted laser desorption/ionization time-of-flight) spectrometry. Genotyping was performed in two Sequenom runs (First: 182 non-IBD, 711 CD; Second: 172 non-IBD, 23 CD). Based on the results of the first genotyping run, additional patients were added for select SNPs which had provided interpretable data.

### Measurement of serum tryptophan, kynurenine and Crohn's disease activity

The kynurenine to tryptophan (K/T) ratio is a reliable method to estimate IDO enzymatic activity which controls for variations in dietary tryptophan intake. [18] We have previously shown that IDO1 expression is increased in active Crohn's disease and that Crohn's disease activity correlates with the serum K/T ratio. [6] High pressure liquid chromatography was used to measure serum tryptophan and kynurenine as previously described by our group. [6] The protocol used was adopted from published reports [19], confirmed for suitability in our environment and tested for assay reliability and reproducibility [(average intra-sample variance of ±4.1% (P = 0.84)]. [6] The K/T ratio was assessed in the serum of patients with IDO1 polymorphisms as compared to patients without genetic polymorphisms and plotted according to clinical disease severity. Crohn's disease severity was categorized using the Physicians Global Assessment (PGA) and confirmed as fitting the American College of Gastroenterology practice guidelines criteria. [20] Patients in symptomatic remission while on corticosteroids were excluded. Serum isolated was from blood samples at the time of acquisition and stored at −80°C until analysis.

### Statistical Analysis

**Figure 1:**
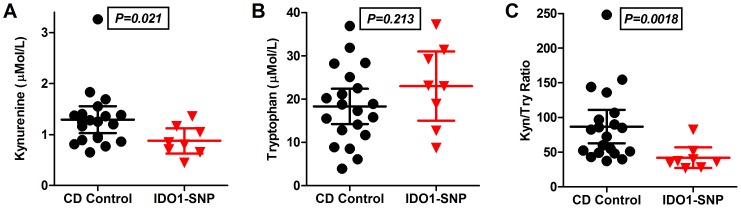
IDO1 activity measurement in Crohn's disease patients with moderate-severely active disease. HPLC was used to measure serum A) Kynurenine and B) Tryptophan from Crohn's disease patients with moderate or severely active Crohn's disease. Controls, CD patients who did not carry SNPs of IDO1 (n = 20), were compared to CD patients who did carry IDO1 SNPs (n = 8). C) The Kyn/Trp ratio, a surrogate of IDO1 activity, was significantly lower in the patients with IDO1 SNPs.

Data analysis and graph assembly was completed using GraphPad Prism (GraphPad Software, La Jolla, CA) and Software R (http://www.r-project.org). Mann-Whitney *U* test was used for non-parametric continuous variables (Fig. 1) and Fisher's exact test for univariate analysis of categorical variables. Mean values are presented with calculated standard deviation in parentheses. To control for multiple testing, the Hochberg method of controlling the false discovery rate (FDR) was also computed using software R to test for significance (FDR of <0.1). [21] Logistic regression based on dichotomous outcomes was used to calculate odds ratio (OR) with 95% confidence intervals (CI) and significance as reported in the text. For the investigated variants, predetermined minor allele frequencies were obtained from HapMap and dbSNP, and experimental minor allele frequencies were computed from our genotyping results. Allele frequencies were compared using the Fisher's exact test. P values of <0.05 were considered statistically significant.

A multiple logistic regression analysis was conducted to access the significance of the association between IDO2 SNPs and CD risk after adjusting for effect from race, gender and age at diagnosis. [22] The regression was fitted with both dominant and recessive models with all races other than white and black grouped together. The baseline level of race and gender was chosen as White and Male respectively. Additionally, to identify if IDO1 SNP carriage is among the factors associated with the CD phenotypes identified on univariate analysis, a logistic regression was fitted with stepwise variable selection based on Akaike information criterion (AIC). [15] The independent variables considered were presence of IDO1 minor allele, race, gender, age at diagnosis and family history. Both the analysis were computed also in software R.

## Results

### Demographics

The total study population included 734 patients in the CD group and 354 in the non-IBD group. The CD group had a lower mean age (44.2 vs 52.5 years; P<.01), included more males (44.6% vs 32.2%; P<.01), and had fewer African Americans (9.3% vs 17.8%; P<.01) in comparison to the non-IBD group. These differences likely reflect the IBD patient population compared to the cohort of individuals that volunteer to participate in open access studies or who are eligible for colorectal cancer screening in the study enrollment region.

### IDO1 Genotyping

A total of eight patients in the CD cohort carried IDO1 genetic variants compared to six individuals in the non-IBD cohort (1.1% vs 1.7%, P = 0.40). Predicted and experimental IDO1 minor allele frequencies are shown in Table 1. RS4463407 and rs12545877 were listed as potential SNPs in dbSNP; however, there was no frequency available in the online databases and we did not identify their presence in our screening evaluation of 182 non-IBD controls and 711 CD patients. For each of the other IDO1 variants, there were no significant differences between proportions of non-IBD and CD patients carrying at least 1 variant allele. This held true when examined by race as well. All patients with the 7CA gene variant demonstrated homozygosity for the variant allele, a finding which nearly reached significance (non-IBD 0/674 vs CD 8/1220; P = 0.057). Minor allele frequencies did not differ between groups for the other variants.

**Table 1:** Expected and identified frequency of patients with IDO1 Variant Alleles.

IDO1 Variant	Predicted Minor Allele Frequencies	Non-IBD Cohort (354 patients)	Crohn's Disease Cohort (734 patients)	P Value
rs4463407 (G to A)	N/A	0/181 (0%)	0/705 (0%)	-
rs12545877 (A to G)	N/A	0/180 (0%)	0/702 (0%)	-
rs35059413 (C to T)	0.7%–4.4%	5/345 (1.45%)	3/716 (0.42%)	0.122
rs35099072 (C to T)	1.4%–4.2%	0/351 (0%)	1/723 (0.14%)	1.000
7CA (Exon 7, C to A)*	0%–1.042%	0/337 (0%)	4/610 (0.66%)	0.303
9BPD (Exon 7, 9 base pair deletion)	0%–1.042%	1/346 (0.29%)**	0/722 (0%)	0.330

Notes: The denominator is shown to indicate the number of successful complete reads per genotype.

*All 7CA variants were homozygous while all other variants were single allele.

**This individual also carried a single allele of the rs25059413 variant.

### IDO2 Genotyping

We next sought to determine if there were any potential differences in the prevalence of IDO2, a gene paralog to IDO1, variants between a CD and non-IBD population. Observed variant minor allele frequencies of IDO2 were common, but fell within the predicted minor allele frequencies (Table 2). We did not identify any individuals with the predicted one base pair deletion variant rs35446289. Adjusting for age of diagnosis, race and gender and using the recessive model, the IDO2 variant rs4503083 was less common in CD patients than non-IBD controls. No significant findings were identified using the dominant model. Interestingly, rs35212142 which had a positive, but non-significant association with CD risk on the adjusted analysis, showed a significant association on univariate analysis for individuals carrying at least one variant allele (OR 1.54, 95% CI 1.1–2.2; P = 0.016). This finding remained significant by multiple testing (FDR = 0.072).

**Table 2:** Expected and identified frequency of patients with IDO2 Variant Alleles.

		Recessive Logistic Model		Number (and %) of homozygous minor allele		
IDO2 Variant	Predicted Minor allele frequency	Estimate coefficient	P-value	Race	Non-IBD (177)	CD (686)
**rs4503083 (T to A)**	**10.9%**–**50.0%**	**−1.295**	**0.025**	**White**	**66/142 (46.5%)**	**399/614 (35.0%)**
**Y359stop**				**Black**	**6/34 (17.6%)**	**13/63 (20.6%)**
				**Other**	**0/1 (0%)**	**7/9 (77.8%)**
rs4736794 (A to G)	6.7%–50.0%	−0.498	0.698	White	29/142 (46.5%)	399/614 (35.0%)
				Black	2/34 (5.9%)	13/63 (3.2%)
				Other	0/1 (0%)	6/9 (66.7%)
rs10109853 (C to T)	18.2%–100%	−0.072	0.786	White	97/142 (68.3%)	473/614 (77.0%)
				Black	20/34 (58.8%)	35/63 (55.6%)
				Other	1/1 (100%)	7/9 (77.8%)
rs35212142(T to A)	5.6%–50.0%	12.923	0.981	White	8/142 (5.6%)	28/614 (4.6%)
				Black	2/34 (5.9%)	0/63 (0%)
				Other	0/1 (0%)	0/9 (0%)

Several patients carried overlapping IDO2 minor allele SNPs. When considering all gene variants, the likelihood of carrying at least one IDO2 SNP was similar between cohorts (81.3% non-IBD vs 84.0% CD; P = 0.43). Moreover, the number of those who were homozygous for at least one IDO2 SNP was not different between non-IBD and CD patients (27.6% vs 25.9%; P = 0.572).

### IDO1 Genotype-Phenotype Correlation in Crohn's Disease

There is strong clinical and preclinical evidence implicating a role for IDO1 in modulating IBD disease activity. [7] For this reason we focused on the clinical characteristics of patients who carried the identified IDO1 SNPs as compared to the CD cohort without any IDO1 variant (Table 3). IDO1 SNP patients were more likely to be African American than those without, a finding predicted based on the online genotype databases. In multiple logistic regression analysis accounting for race, gender, age of onset and family history we found IDO1 SNP carrying patients more likely to have extraintestinal manifestations of disease (P = 0.005) and perianal involvement (P = 0.01).

**Table 3:** IDO1 Variants and Crohn's Disease Phenotype.

Characteristic	CD Group without any IDO1 Variant (726 patients)	CD Group with any IDO1 Variant (8 patients)	P Value
Mean Age at Diagnosis in years (SD)	28.28 (13.00)	30.63 (12.53)	0.614
Age Range at Diagnosis in years	1–77	17–50	
Gender			
Male (%)	324 (44.63%)	4 (50.00%)	0.737
Female (%)	402 (55.37%)	4 (50.00%)	0.737
Race			
** White (%)**	**651 (89.67%)**	**3 (37.50%)**	**<0.001**
** Black (%)**	**63 (8.68%)**	**5 (62.50%)**	**<0.001**
Other (%)	12 (1.65%)	0	1.000
Family History of IBD (%)			
No	563 (77.5%)	6 (75%)	1.000
First degree	86 (11.8%)	1 (12.5%)	
Second degree	70 (9.6%)	1 (12.5%)	
Unknown	7 (1.0%)		
History of IBD-Related Surgery (%)			
One surgery	484 (66.7%)	8 (100.00%)	0.058
Two surgeries	211 (29.1%)	4 (50%)	
> 2 surgeries	89 (12.3%)	2 (25%)	
Use of Steroid Sparing Medication	453 (62.4%)	8 (100%)	0.0288
**Extra Intestinal Manifestations (%)**	**169 (23.28%)**	**6 (75.00%)**	**0.0031** *
Montreal Disease Classification			
Location			
L1: Terminal ileum or cecum only (%)	313 (43.11%)	2 (25.00%)	0.477
L2: Colon only (%)	152 (20.94%)	1 (12.50%)	1.000
L3: Terminal ileum and colon (%)	256 (35.26%)	5 (62.50%)	0.141
L4: Upper small bowel only (%)	5 (0.69%)	0	1.000
Upper Small Bowel Involvement (%)	88 (12.12%)	1 (12.50%)	1.000
Disease Behavior			
B1: Inflammatory (%)	238 (32.78%)	3 (37.50%)	0.722
B2: Stricturing (%)	198 (27.27%)	3 (37.50%)	0.691
B3: Penetrating (%)	290 (39.94%)	2 (25.00%)	0.488
**Perianal Involvement (%)**	**209 (28.79%)**	**6 (75.00%)**	**0.0096** *

*These values remained significant in univariate analysis when comparing within black race only. EIMs: 15/63 vs 5/5, P = 0.002; Perianal involvement: 20/63 vs 4/5, P = 0.049 Use of steroid sparing medication did not meet significance when comparing within black race only: 31/63 vs 5/5, P = 0.056.

To validate the multivariate comparisons, we performed univariate analysis and found extraintestinal disease manifestations (EIM) including IBD-associated arthritis and uveitis were more common in IDO1 SNP patients (OR 9.89, CI 2.0–49.4 with P = 0.005 and FDR = 0.019). Perianal involvement, an indicator of disease severity, also more commonly affected the SNP carrying cohort (OR 7.42, CI 1.5–37.1 with P = 0.015 and FDR = 0.046). While race could be considered an influential factor in these phenotypes, both results remain significant on univariate analysis when comparing within African Americans only (see table footnote).

Individual patient characteristics are described in Table 4 and highlight a generally severe clinical course marked by ubiquitous need for surgery(s) and steroid-sparing therapies (azathioprine/mercaptopurine/methotrexate/TNF-α inhibitors). Use of steroid sparing medications was significantly higher in the IDO1 SNP cohort as a whole, but not when subcategorized by race or when taking into account multiple testing (FDR = 0.109).

**Table 4:** Specific Phenotypic Data of Individual Patients with at Least 1 IDO1 Variant Allele.

Variant	Genotype	Sex	Race	Age at Dx	Family History	Surgery (N)	Montreal Location	Perianal Disease	Montreal Behavior	Extraintestinal Manifestations	Use of IMM/TNFα
rs35059413	AG	M	Black	30	Yes	Yes (2)	L3	Yes	B3	Yes	Yes
rs35059413	AG	F	Black	17	No	Yes (6)	L3	Yes	B3	Yes	Yes
rs35059413	AG	M	Black	22	No	Yes (1)	L2	Yes	B1	Yes	Yes
rs35099072	CA	F	Black	43	No	Yes (1)	L1	No	B1	Yes	Yes
Exon 7, C to A	AA	F	White	41	Yes	Yes (3)	L1#	No	B2	No	Yes
Exon 7, C to A	AA	M	Black	50	No	Yes (1)	L3	Yes	B2	Yes	Yes
Exon 7, C to A	AA	F	White	18	No	Yes (2)	L3	Yes	B1	No	Yes
Exon 7, C to A	AA	M	White	23	No	Yes (1)	L3	Yes	B2	Yes	Yes

# Denotes upper small bowel involvement in addition to other location.

Abbreviations: IMM, immunomodulators; TNFα, Antibodies against Tumor Necrosis Factor alpha.

### SNPs and IDO1 enzymatic activity

We further sought to examine whether IDO1 SNPs were associated with detectable differences in enzyme function in vivo. All patients with IDO1 SNPs had serum sampled during a period of moderate to severe Crohn's disease activity. Serum tryptophan and kynurenine levels were determined using an established method and compared to CD patients without IDO1 variants during a time of similar disease activity. Fig. 1 shows that while tryptophan levels were similar, kynurenine and the K/T ratio were significantly lower in the IDO1 variant cohort. The K/T ratio is considered an accurate surrogate for IDO1 enzyme activity; thus these data suggest that functional impairment of enzymatic activity may account for the observed IDO1 variant genotype-phenotype associations.

### IDO2 Genotype-Phenotype Correlation in Crohn's Disease

IDO2 variants were more common among White than African Americans. Penetrating disease was less common in patients with any homozygous IDO2 SNP on univariate analysis. However, this finding did not stay significant on multiple testing and no other clinical characteristics were identified to be different when comparing patients with no identified SNPs to those who were either heterozygous or homozygous for IDO2 SNPs (Table 5). The same was true for each SNP considered independently (data not shown). These data suggest that IDO2, unlike IDO1, does not influence CD clinical phenotype.

**Table 5:** IDO2 Variants and Crohn's Disease Phenotype.

Patient Characteristic	No IDO2 SNP (114 patients)	Any IDO2 SNP (heterozygous and homozygous) (597 patients)	P Value*	Any homozygous IDO2 SNP (184 pts)	P Value*
Mean Age at Diagnosis in years (SD)	27.70 (12.10)	28.38 (13.14)		27.5 (12.01)	0.98
Age Range at Diagnosis in years	5–71	1–77		4–77	
Gender					
Male (%)	50 (43.86%)	267 (44.72%)	0.92	80(43.4%)	1.00
Female (%)	64 (56.14%)	330 (55.28%)	0.92	104(56.5%)	1.00
Race					
** White (%)**	**90 (78.95%)**	**545 (91.29%)** *	**<0.001**	**172(93.4%)**	**<0.001**
** Black (%)**	**23 (20.18%)**	**44 (7.37%)** *	**<0.001**	**11(5.9%)**	**<0.001**
Other (%)	1 (0.88%)	8 (1.34%)	1.00	1(0.5%)	1.00
Family History of IBD (%)			1.00		0.66
No	89 (78.1%)	461 (77.2%)		148 (80.4%)	
First degree	11 (9.6%)	76 (12.7%)		23 (12.5%)	
Second degree	14 (12.3%)	53 (8.9%)		12 (6.5%)	
Unknown		7 (1.2%)		1 (0.5%)	
History of IBD-Related Surgery (%)			0.66		0.53
One surgery	79 (69.30%)	398 (66.33%)		120 (65.2%)	
Two surgeries	29 (25.43%)	186 (31.15%)		54 (29%)	
>2 surgeries	12 (10.5%)	79 (13.23%)		25 (13.5%)	
Use of Steroid Sparing Medication	75 (65.8%)	383 (64.2%)	0.83	125 (67.9%)	0.71
Extra Intestinal Manifestations (%)	28 (24.56%)	139 (23.28%)	0.81	45(24.4%)	1.00
Montreal Disease Classification					
Location					
L1: Terminal ileum or cecum only (%)	51 (44.74%)	263 (44.05%)	0.92	90 (48.9%)	0.55
L2: Colon only (%)	22 (19.30%)	127 (21.27%)	0.71	43 (23.4%)	0.47
L3: Terminal ileum and colon (%)	41 (35.96%)	202 (33.84%)	0.67	51 (27.7%)	0.16
L4: Upper small bowel only (%)	0	5 (0.84%)	1.00	0	1.00
Upper Small Bowel Involvement (%)	16 (14.04%)	64 (10.72%)	0.33	13 (7.1%)	0.069
Disease Behavior					
B1: Inflammatory (%)	33 (28.95%)	200 (33.50%)	0.38	70 (38.0%)	0.133
B2: Stricturing (%)	30 (26.32%)	167 (27.97%)	0.82	53 (28.8%)	0.691
B3: Penetrating (%)	51 (44.74%)	230 (38.53%)	0.25	61 (33.2%)	0.0496#
Perianal Involvement (%)	30 (26.32%)	179 (29.98%)	0.50	41 (22.3%)	0.48

*P values versus patient cohort with no IDO2 SNP.

# Not significant on multivariate analysis.

## Discussion

Utilizing a prospectively collected database of clinically phenotyped IBD patients, we genotyped for established IDO1 and IDO2 SNPs and novel genetic variants elucidated in the literature. IDO1 SNPs were rare and not linked to Crohn's disease risk in this population. However, they were associated with a severe clinical course, presence of perianal disease, extraintestinal manifestations and a reduced serum K/T ratio during active disease. IDO2 SNPs were common and varied in frequency according to race and CD risk. No IDO2 SNPs associated with a particular Crohn's disease clinical phenotype. This work highlights the functional importance of IDO enzymes in human Crohn's disease and establishes relative rates of IDO genetic variants in a large US population.

The finding that one of the IDO2 minor allele variants conferred CD risk is provocative, particularly given that this SNP, rs10109853 (R248W), confers a 90% reduction in enzymatic activity. [23] However, IDO2 SNPs also varied by race, and the effect was lost when analyzed taking into account multiple variables including race. Interesting, this multiple analysis then demonstrated a protective effect against CD for individuals with homozygous IDO2 variant rs4500383. While its expression in the colon is reported, the understanding of IDO2's relevance to human health, and especially gastrointestinal disease, is less developed than that of IDO1. [7], [9] Most functional investigations addressing IDO2 have focused on its expression in professional antigen presenting cells (pAPCs) and potential as a target for cancer immunotherapy. [24] As pAPCs also mediate critical roles in mucosal immune tolerance and IBD [25], this provides a plausible mechanistic explanation and underscores the potential importance of our findings. Additionally, recent reports suggests that important interactions may occur between IDO1 and IDO2. [26], [27] Clearly our findings implicating IDO2 in CD risk will require confirmation in a larger CD and control cohort as the overall prevalence of IDO2 SNPs are high and vary by race.

Extra-intestinal manifestations and perianal disease were both more common in IDO1 SNP carrying patients. Though overlap exists between the risk alleles for IBD and other inflammatory diseases, very little is known as to whether any of these polymorphisms confer risk for IBD associated extra-intestinal manifestations. The finding that IBD associated arthritis and uveitis occurred in patients with IDO1 SNPs is supported by studies linking elevated IDO1 expression to disease activity and pathogenesis in both inflammatory arthritis [28] and experimental uveitis. [29] Additionally, the tie of IDO1 to perianal disease is intriguing as the roles for IDO1 expressing fibroblasts and epithelial cells is just beginning to be addressed [10], [30], [31].

The impact of IDO1 polymorphisms on enzymatic activity was assessed using the K/T ratio, a well-established surrogate for IDO1 function. We found that in composite, non-synonymous IDO1 SNPs conferred a diminishment in enzymatic activity (mean K/T of 42.1 vs 86.9 for non-IDO1 SNP CD patients). This average was numerically higher, though not statistically different from the K/T of 35.1 in a non-CD control cohort of patients examined previously by our group. [6] While this is the first study to examine the impact on human samples, our findings are supported by previous work using computational prediction analysis and cell transfection experiments. [14] As IDO1 may be susceptible to functionally relevant post-translational modification [32], future studies directed at confirming this effect should target functional assays using freshly cultured cells from SNP carrying individuals.

Sample size is a significant study limitation especially in comparison to larger genome wide association studies. As IDO1 SNPs are rare, a larger cohort would be required to assess the association of disease risk. Using a case:control ratio of 1∶5, the determination of a 50% increase in risk could be achieved by including between 552 and 2985 cases (based on allelic frequency ranging from 4.4% down to 0.7% respectively), assuming 80% power and Type 1 error rate of 0.05. [33] Sequenome genotyping efficiency was also not 100%, thus our experimentally identified rates of variance may slightly over or underestimate true population prevalence. Despite these limitations, this is the largest study to date to examine a disease and control population for IDO1 and IDO2 SNPs. As such, our work adds to recent studies which have examined the links between IDO gene polymorphisms and disease including pre-eclampsia [34] and systemic sclerosis [35] for IDO1, and, for IDO2, relevance in pancreatic cancer [36] and clinical response to antidepressants [37].

In summary, our data newly link IDO SNPs and minor allele variants to Crohn's disease risk and phenotype. As IDO1 variants are modestly rare, large disease and control cohorts will need to be evaluated to identify if these SNPs confer increased disease risk or associate with less common disease phenotypes. Moreover, confirmative studies should be extended to include an ulcerative colitis cohort. Finally, if therapies directed at enhancing or blocking IDO function move from early phase [38], [39] to phase 3 clinical trials, our data would suggest that IDO SNP genotyping may be important in establishing enrollment.
