# Correction: IDO1 and IDO2 Non-Synonymous Gene Variants: Correlation with Crohn's Disease Risk and Clinical Phenotype

**DOI:** 10.1371/journal.pone.0133098

**Published:** 2015-07-14

**Authors:** Alexander Lee, Navya Kanuri, Yuanhao Zhang, Gregory S. Sayuk, Ellen Li, Matthew A. Ciorba

Table 2 is incorrect. Please see the corrected Table 2 here.

**Table 2:** Expected and identified frequency of patients with IDO2 Variant Alleles.

		Recessive Logistic Model		Number (and %) of minor alleles		
	Predicted Minor allele frequency	Estimate coefficient	P-value	Race	Non-IBD (N = 177)	CD (N = 686)
**rs4503083 (T to A)**	**10.9%–50.0%**	**-1.295**	**0.025**	**White**	**het: 58 (40.8%)**	**het: 196 (31.9%)**
**Y359stop**					**homo: 8 (5.6%)**	**homo: 19 (3.1%)**
					**total: 142**	**total: 614**
				**Black**	**het: 5 (14.7%)**	**het: 12 (19%)**
					**homo: 1 (2.9%)**	**homo: 1 (1.6%)**
					**total: 34**	**total: 63**
				**Other**	**het: 0 (0%)**	**het: 7 (77.8%)**
					**homo: 0 (0%)**	**homo: 0 (0%)**
					**total: 1**	**total: 9**
rs4736794 (A to G)	6.7%–50.0%	-0.498	0.698	White	het: 27 (19%)	het: 92 (15%)
					homo: 2 (1.4%)	homo: 4 (0.7%)
					total: 142	total: 614
				Black	het: 2 (5.9%)	het: 2 (3.2%)
					homo: 0 (0%)	homo: 0 (0%)
					total: 34	total:63
				Other	het: 0 (0%)	het: 6 (66.7%)
					homo: 0 (0%)	homo: 0(0%)
					total: 1	total: 9
rs10109853 (C to T)	18.2%–100%	-0.072	0.786	White	het: 65 (45.8%)	het: 316 (51.5%)
					homo: 32 (22.5%)	homo: 157 (25.6%)
					total: 142	total: 614
				Black	het: 13 (38.2%)	het: 26 (41.3%)
					homo: 7 (20.6%)	homo: 9 (14.3%)
					total: 34	total: 63
				Other	het: 0 (0%)	het: 6 (66.7%)
					homo: 1 (100%)	homo: 1 (11.1%)
					total: 1	total: 9
rs35212142(T to A)	5.6%–50.0%	12.923	0.981	White	het: 8 (5.6%)	het: 26 (4.2%)
					homo: 0 (0%)	homo: 2 (0.3%)
					total: 142	total: 614
				Black	het: 2 (5.9%)	het: 0 (0%)
					homo: 0 (0%)	homo: 0 (0%)
					total: 34	total: 63
				Other	het: 0 (0%)	het: 0 (0%)
					homo: 0 (0%)	homo: 0 (0%)
					total: 1	total: 9
